# Jejunal obstruction due to a variant of transmesocolic hernia: a rare presentation of an acute abdomen

**DOI:** 10.1186/s12893-015-0051-z

**Published:** 2015-05-08

**Authors:** Duminda Subasinghe, Chathuranga Tisara Keppetiyagama, Dharmabandhu N Samarasekera

**Affiliations:** General Surgery, University Surgical Unit, The National Hospital of Sri Lanka, Colombo, Sri Lanka; Gastrointestinal Surgery, University Surgical Unit, The National Hospital of Sri Lanka, Colombo, Sri Lanka; University Surgical Unit, The National Hospital of Sri Lanka, 28/1, Ishwari road, Colombo 06 Colombo, Sri Lanka

**Keywords:** Internal hernia, Transmesocolic, Intestinal obstruction

## Abstract

**Background:**

Internal hernias include paraduodenal, pericecal, through foramen of Winslow, intersigmoid and retroanastomotic hernias. These hernias could be either congenital or acquired after abdominal surgery. They account for approximately 0.5-5 % of all cases of intestinal obstruction.

**Case presentation:**

A 48-year-old female was admitted to casualty with a history of abdominal distension and vomiting of 3 days duration. An abdominal X-ray supine film showed multiple small bowel loops with air fluid levels. On surgery she was found to have a transmesocolic hernia. The defect in the transverse mesocolon was repaired.

**Conclusion:**

The clinical signs and symptoms of lesser sac hernia are non-specific. These rare lesser sac hernias can be lethal. Therefore, immediate diagnosis and surgery is essential. Although a rare entity, they account for significant mortality form intestinal obstruction. We report an extremely rare case of an internal abdominal hernia through the transverse mesocolon, in a young woman.

## Background

Internal hernia is protrusion of a viscus or part of a viscus through anatomical or pathological opening within the limits of peritoneal cavity. They could be either congenital or acquired. There are several main types of internal hernias based on the location as described by Meyers [1]. Specifically these include paraduodenal, pericecal, foramen of Winslow, transmesocolic, inter sigmoid and retroanastomotic hernias. Although the overall incidence of internal hernias are low (0.2–0.9 %) and they accounts only for 0.5 %–5 % of cases of intestinal obstruction, the overall mortality exceeds 50 % if strangulation is present [2, 3]. Transmesocolic hernia is an extremely rare type of internal hernia. Transmesocolic hernia accounts for approximately 5–10 % of all internal hernias [4]. The defects of the mesentery are mostly due to congenital, surgical, traumatic, inflammatory or idiopathic in origin. Although a rare entity, they account for significant mortality form intestinal obstruction. Usually these are detected during surgery for acute abdomen or during an autopsy [5].

## Case presentation

We report a case of transmesocolic herniation of jejunal loops into supracolic compartment with intestinal obstruction which was diagnosed intraoperatively.

**Fig. 1:**
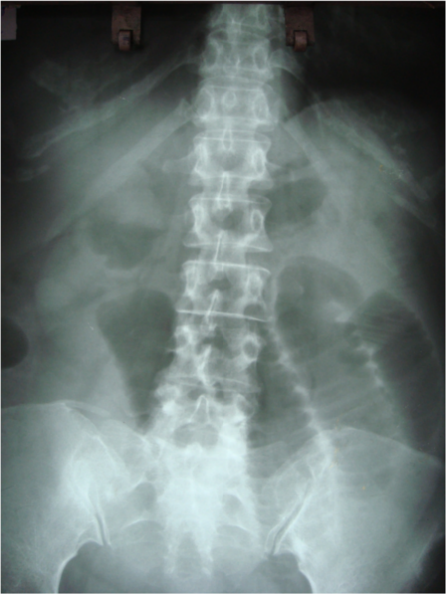
Dilated jejunal loops on X ray abdomen supine film

**Fig. 2:**
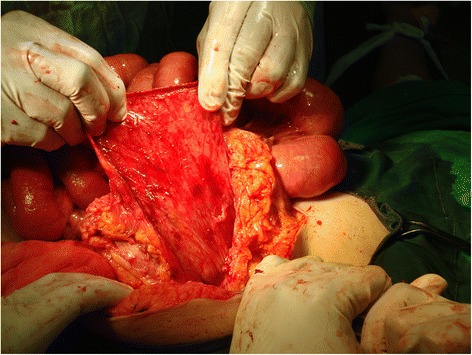
Sac of the transmesocolic hernia

**Fig. 3:**
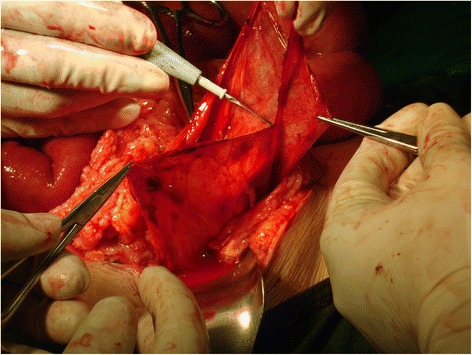
opening and repair of the hernia sac in the supracolic compartment

**Fig. 4:**
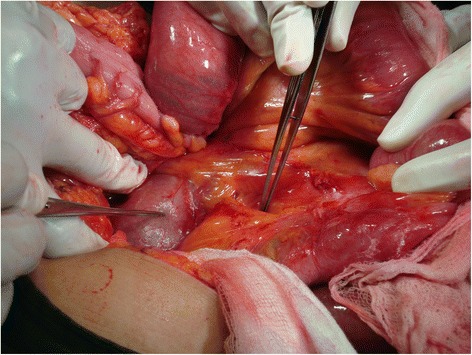
Paraduodenal fossa

**Fig. 5:**
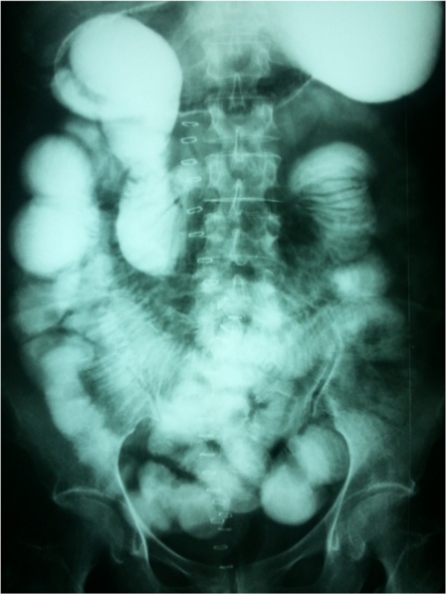
Post operative barium meal and follow through showing normal small intestines

A 48-year-old female was admitted to casualty with a history of abdominal distension and vomiting of 3 days duration. She had no past history of any gastrointestinal surgery but had undergone lower segment caesarean section 21 years earlier. The caesarean section was uneventful without any iatrogenic injury. On admission, she had bilious vomiting. Physical examination revealed tachycardia, generalized abdominal distension, rebound tenderness and rigidity over left upper quadrant. There was no evidence of organomegaly or free fluid and her external hernia orifices were normal. Her bowel sounds were sluggish. Digital rectal examination revealed an empty rectum. Laboratory investigation on admission revealed a normal full blood count with a white blood cell count of 5000/mm3 and normal renal and liver functions. Her serum potassium on admission was 3.5 mmol/l and she was started in intravenous potassium supplements. An abdominal X-ray supine film showed multiple small bowel loops with air fluid levels without free air under the dome of the diaphragm (Fig. 1). Surgical exploration revealed significant amount of free fluid in the peritoneal cavity and ischemic small intestine. On further exploration, we found the DJ flexure in the supracolic compartment and almost all the jejunum and proximal ileum herniating through a small defect about 5 × 6 cm in the transverse mesocolon. Jejunal loops were contained inside a thick walled hernial sac (Fig. 2) which was extending in to the supracolic compartment. The hernia sac with contents was extending into the lesser sac. The contents were reduced and the sac was opened and repaired (Fig. 3). Paraduodenal fossae were found to be normal during the surgery (Fig. 4). The defect in the transverse mesocolon was repaired. Small bowel showed features of viability and therefore, was not resected. The patient was discharged on post operative day 14. Her post operative period was uneventful. She also underwent a contrast study of the small bowel at post op day 10 which showed normal small intestine (Fig. 5).

## Discussion and conclusion

The clinical signs and symptoms of lesser sac hernia are non-specific and include abdominal pain, nausea, vomiting and distension. These rare lesser sac hernias can be lethal. Therefore, immediate diagnosis and surgery is essential. In the literature, only few cases of internal hernias have been documented [6]. The anomaly of transmesocolic herniation, which was first reported by Rokitansky in 1836 is an extremely rare type of internal hernia [2]. According to the literature, herniation into the lesser sac can be classified into three basic types according to the site of the aperture [7, 8]. Type 1 is a hernia through the foramen of Winslow, type 2 is a hernia through a defect in the lesser or greater omentum and type 3 is a hernia through a defect in the transverse mesocolon. Our patient had type 3 transmesocolic hernia. Type 3 is usually secondary to abdominal trauma or prior abdominal surgery with the creation of a Roux-en-Y loop [9, 10]. Approximately 5–10 % of all internal hernias occur through defects in the mesentery of the small bowel and almost 35 % of transmesocolic hernias are observed among paediatric age group, mainly those aged between 3 and 10 years [3]. In adults, however most mesenteric defects are the result of previous gastrointestinal operations, abdominal trauma or intra peritoneal inflammation [11–13]. Our case was a rare presentation in an adult without a history of trauma or previous bowel surgery. Gomes et al. [3] and described a patient with congenital transmesenteric type internal hernia presented with intractable colick epigastric pain. Frediani et al. [6] has described a transmesocolic hernia presented with small intestinal obstruction. Agresta et al. [4] has described two patients presented with acute small intestinal obstruction due to internal hernia during immediate post operative period following laparoscopic hernia repair.

Although tansmesocolic hernia is a difficult preoperative diagnosis, CT abdomen might help the diagnosis by peripherally located small bowel, and lack of omental fat between the loops and the anterior abdominal wall [14, 15]. Congenital tansmesocolic hernias are extremely rare and todate only few cases of transmesocolic hernias were reported in the literature [3, 6, 16].

In conclusion, diagnosis of intestinal obstruction caused by a congenital mesocolic hernia remains difficult preoperatively despite the techniques currently available, so it is important to consider the possibility of a transmesocolic hernia in a patient with ileus even with no past history of gastrointestinal surgery.

### Consent

Written informed consent was obtained from the patient for publication of this case report and any accompanying images. A copy of the written consent is available for review by the Editor-in-Chief of this journal.
